# Association Between the Time of Surgery and the Incidence of Postoperative Nausea-Vomiting: A Propensity Score Matched Analysis

**DOI:** 10.7759/cureus.72737

**Published:** 2024-10-30

**Authors:** Shariq A Khan, Hsiang Lim, Shivani Harikrishnan, Harikrishnan Kothandan

**Affiliations:** 1 Anaesthesia, Sengkang General Hospital, Singapore, SGP; 2 Biostatistics, Singapore General Hospital, Singapore, SGP; 3 Anesthesiology, Nottingham City Hospital, Nottingham, GBR; 4 Anaesthesiology and Perioperative Medicine, Singapore General Hospital, Singapore, SGP

**Keywords:** anaesthesia, circadian cycle, incidence of postoperative nausea and vomiting, post-operative nausea and vomiting (ponv), postoperative outcomes

## Abstract

Background

The probability of postoperative nausea and vomiting (PONV) may be influenced by the time of day. Since there has been no recent study published exploring this association, we conducted a study to determine the effect of surgical time of day on PONV.

Methods

With ethics approval, data of all patients undergoing elective non-cardiac non-neurosurgery, with a high risk of PONV, and which had a 24-hour assessment of PONV between October 2017 and December 2020 were extracted. Surgical sessions were classified as either morning surgery (defined as start-time and end-time before noon) or afternoon surgery (defined as surgery start-time at or after 12 noon). Those patients who had surgical sessions extending from morning sessions to afternoon sessions were excluded. A propensity-score-matching (PSM) based on 1:1 matching was performed to potential biases. For the entire study population before matching, we used Pearson’s chi-square test or Fisher’s exact test for categorical variables, and Student’s t-test or Mann-Whitney U-test for normally or non-normally distributed continuous variables, respectively. For the matched study population, we used conditional logistic regression to test for differences. A p-value of <0.05 was considered statistically significant.

Results

Of the 3808 surgical sessions, 1439 surgical sessions were excluded from the analysis because they extended between morning and afternoon surgery. After 1:1 PSM, 1043 matched pairs were obtained. The incidence of PONV, prior to matching, remained statistically insignificant even after PSM.

Conclusion

We found that the time of surgery (morning or afternoon) did not affect both the overall incidence and severity of PONV. The association between PONV and time of day remained statistically insignificant prior to and after propensity matching for a number of covariates.

## Introduction

Several studies have shown that the time of surgery may affect the incidence of postoperative complications. It has been postulated that late work-day surgery has higher associated postoperative complications because of factors like unavailability of personnel and hospital resources or provider fatigue [1,2]. Some studies have also postulated that changes in biological processes governed by circadian rhythms may affect susceptibility to complications at different times of day [3-6].

Postoperative nausea and vomiting (PONV) is a common adverse event following surgery, occurring in as much as 80% of some high-risk patients [7,8]. PONV not only causes patients significant distress but also reduces overall satisfaction [9], and increases the risk of postoperative complications and healthcare costs [10]. A study by Wright et al. [2] found that the predicted probability of postoperative adverse events like pain and PONV increased from 1% at 9 am to 4.2% at 4 pm and the probability of PONV was increased for cases starting at 2 pm compared with 7 am. The authors concluded that it was unclear whether the time of day effects on adverse events like PONV were due to factors like care transition periods, care provider fatigue or physiological changes in patients and went on to suggest further research in this area. To the best of our knowledge, there has been no study published exploring the association between the time of surgery and the incidence of PONV since the study by Wright et al. To address this gap in knowledge, we analysed data from our hospital perioperative database to assess whether the time of surgery (morning vs. afternoon) affects the incidence (primary outcome) and severity (secondary outcome) of postoperative nausea and vomiting (PONV) in high-risk patients undergoing elective non-cardiac, non-neurosurgical procedures. (This article was previously presented as a poster at the 2023 European Society of Regional Anesthesia Meeting on October 10, 2023)

## Materials and methods

After obtaining institutional review board approval (Singhealth CIRB: 202207-00006), perioperative data of all patients undergoing elective non-cardiac non-neurosurgery, with a high risk of PONV (defined as patients with a preoperative Apfel score [8] of 3 or more undergoing an >1-hour duration surgery under general anaesthesia) and which had a 24-hour postoperative assessment of PONV between October 2017 and December 2020 at Singapore General Hospital was extracted for analysis from the SingHealth-IHiS Electronic Health Intelligence System (eHints). eHints is an award-winning system which features a single enterprise data repository that integrates information from multiple healthcare transactional systems including Administration, Clinical and Ancillary systems. This provides healthcare staff with highly integrated and quality information. The improved analytical tools enable users to perform 'self-service' reporting and analysis [11].

Surgical sessions were classified as either morning surgery (defined as surgery start time and end time before 12 noon) or afternoon surgery (defined as surgery start time at or after 12 noon) for analysis. Those patients who had surgical sessions extending from morning sessions to afternoon sessions were excluded from the analysis.

We studied the association between surgical session time of day (Morning or Afternoon Session) and the incidence of PONV (Primary Outcome). A study subject was defined to have PONV if the 24-hour postoperative assessment record mentioned the presence of nausea, vomiting or both. At our hospital, it is the standard of care for all elective patients to be reviewed within 24 hours postoperatively either in person at the ward or by phone call (if discharged) by a member of the anaesthesia team (PONV is scored as ‘nil’, ‘mild’ if only nausea episodes, ‘moderate’ if there are <3 vomiting episodes, or ‘severe’ if there are >=3 vomiting episodes in the postoperative period).

A propensity score matching based on 1:1 matching was performed to potential biases between the morning and afternoon surgery groups. The propensity scores were estimated using multivariable logistic regression with covariates which were found to be significantly different between the unmatched groups on univariate analysis (namely, session duration, surgical specialty, age at time of surgery, and intraoperative anti-emetics). This was performed using psmatch2 in Stata. The balance of the covariates was assessed before and after matching through assessment of the standardised mean differences in the covariates and propensity score in the unmatched and matched groups.

Descriptive statistics were summarised using mean (standard deviation; SD), median (range), or frequency (percentage) as appropriate. We assessed for normality using the Shapiro-Wilk test. For the entire study population before matching, we used Pearson’s chi-square test or Fisher’s exact test for categorical variables, and Student’s t-test or Mann-Whitney U test for normally or non-normally distributed continuous variables, respectively to test for differences in patient characteristics between the morning and afternoon surgery groups. For the matched study population, we used conditional logistic regression to test for differences. A 2-sided p-value of <0.05 was considered statistically significant. All statistical analyses were performed using Stata 17 (Stata Statistical Software: Release 17, 2021, StataCorp LLC, College Station, TX, USA).

## Results

Of the 3808 surgical sessions that met the inclusion criteria, 1439 surgical sessions were excluded from the analysis because they extended between morning and afternoon surgery. Prior to propensity score matching, morning surgical session had a significantly higher incidence of use of intraoperative antiemetic combinations of ondansetron and dexamethasone, significantly lower age and shorter duration of surgery and lower use of total intravenous anesthesia (TIVA) than afternoon surgical sessions (Table 1). After 1:1 propensity score matching, 1043 matched pair of morning and afternoon surgical session was obtained (Table 2).

**Table 1:** Summary of baseline patient characteristics (UNMATCHED). Values are in number (proportion), mean (SD) or median (IQR (range)). ENT: Ear, nose and throat surgery, O&G: Obstetrics and gynecology

	Before matching		
	Morning surgery (n=1043)	Afternoon surgery (n=1326)	p-value
Age at time of surgery	58.0 (14.7)	59.6 (14.2)	0.005
Gender			
Male	23 (2.2)	19 (1.4)	0.16
Female	1020 (97.8)	1307 (98.6)	
Race			
Chinese	785 (75.3)	994 (75.0)	0.87
Malays	98 (9.4)	125 (9.4)	
Indians	78 (7.5)	110 (8.3)	
Others	82 (7.9)	97 (7.3)	
Apfel Score			
3	964 (92.4)	1236 (93.2)	0.46
4	79 (7.6)	90 (6.8)	
Session duration	110 (85-145 (60-235))	120 (85-161 (60-237))	0.0008
Laparoscopic surgery			
No	1002 (96.1)	1270 (95.8)	0.72
Yes	41 (3.9)	56 (4.2)	
Number of intraoperative AntiEmetics used			
0	76 (7.3)	147 (11.1)	
1	371 (35.6)	518 (39.1)	0.001
2	584 (56.0)	646 (48.7)	
3	12 (1.2)	15 (1.1)	
Intraoperative Dexamethasone	4 (0-8 (0-32))	4 (0-8 (0-32))	0.16
Intraoperative Ondansetron	4 (4-4 (0-32))	4 (4-4 (0-32))	0.008
Intraoperative Fentanyl	100 (50-100 (0-1000))	100 (50-100 (0-900))	0.14
Intraoperative Morphine	5 (0-8 (0-30))	4.5 (0-7 (0-32))	0.07
Intraoperative Metoclopramide	0 (0-0 (0-20))	0 (0-0 (0-20))	0.87
Intraoperative Ondansetron and Dexamethasone use	593 (56.9)	658 (49.6)	<0.001
Anaesthesia Type			
Total intravenous anaesthesia	120 (11.5)	210 (15.8)	0.003
Gas anaesthesia	923 (88.5)	1116 (84.2)	
Nitrous oxide use	217 (20.8)	288 (21.7)	0.59
Specialty			
ENT	54 (5.2)	75 (5.7)	<0.001
General Surgery	330 (31.6)	306 (23.1)	
O&G	159 (15.2)	159 (12.0)	
Orthopaedics	412 (39.5)	678 (51.1)	
Plastic	23 (2.2)	25 (1.9)	
Urology	33 (3.2)	49 (3.7)	
Others	32 (3.1)	34 (2.6)	

**Table 2:** Summary of baseline patient characteristics (MATCHED). Values are in number (proportion), mean (SD) or median (IQR (range)). ENT: Ear, nose and throat surgery, O&G: Obstetrics and gynecology

	After matching		
	Morning surgery (n=1043)	Afternoon surgery (n=1043)	p-value
Age at the time of surgery	58.0 (14.7)	57.5 (14.1)	0.43
Gender			
Male	23 (2.2)	14 (1.3)	0.14
Female	1020 (97.8)	1029 (98.7)	
Race			
Chinese	785 (75.3)	769 (73.7)	0.75
Malays	98 (9.4)	97 (9.3)	
Indians	78 (7.5)	90 (8.6)	
Others	82 (7.9)	87 (8.3)	
Apfel Score			
3	964 (92.4)	970 (93.0)	0.62
4	79 (7.6)	73 (7.0)	
Session duration	110 (85-145 (60-235))	105 (80-155 (60-237))	0.66
Laparoscopic surgery			
No	1002 (96.1)	991 (95.0)	0.24
Yes	41 (3.9)	52 (5.0)	
Number of intraoperative AntiEmetics used			
0	76 (7.3)	82 (7.9)	
1	371 (35.6)	346 (33.2)	0.60
2	584 (56.0)	600 (57.5)	
3	12 (1.2)	15 (1.4)	
Intraoperative Dexamethasone	4 (0-8 (0-32))	4 (0-8 (0-32))	0.24
Intraoperative Ondansetron	4 (4-4 (0-32))	4 (4-4 (0-32))	0.88
Intraoperative Fentanyl	100 (50-100 (0-1000))	100 (50-100 (0-900))	0.85
Intraoperative Morphine	5 (0-8 (0-30))	5 (0-8 (0-32))	0.56
Intraoperative Metoclopramide	0 (0-0 (0-20))	0 (0-0 (0-20))	0.99
Intraoperative Ondansetron and Dexamethasone use	593 (56.9)	614 (58.9)	0.34
Anaesthesia Type			
Total Intravenous Anaesthesia	120 (11.5)	120 (11.5)	0.99
Gas Anaesthesia	923 (88.5)	923 (88.5)	
Nitrous oxide use	217 (20.8)	251 (24.1)	0.07
Specialty			
ENT	54 (5.2)	59 (5.7)	0.90
General Surgery	330 (31.6)	306 (29.3)	
O&G	159 (15.2)	159 (15.2)	
Orthopaedics	412 (39.5)	435 (41.7)	
Plastic	23 (2.2)	21 (2.0)	
Urology	33 (3.2)	29 (2.8)	
Others	32 (3.1)	34 (3.3)	

There was no significant difference between the morning surgery and afternoon surgery group after matching. The incidence of PONV, prior to matching (12.3% and 14.0%, in the morning surgery group and afternoon surgery groups, respectively) remained statistically insignificant even after propensity score matching (12.3% and 14.4%, in the morning and afternoon surgery groups, respectively) (Table 3).

**Table 3:** Differences in postoperative nausea and vomiting between AM and PM surgery patients. Values are in number (proportion). PONV: Postoperative nausea and vomiting

	Before matching			After matching		
Variable	Morning surgery	Afternoon surgery	p-value	Morning surgery	Afternoon surgery	p-value
PONV	128 (12.3)	186 (14.0)	0.21	128 (12.3)	150 (14.4)	0.17
PONV grade						
Nil	915 (87.7)	1140 (86.0)	0.30	915 (87.7)	893 (85.6)	0.57
Mild	105 (10.1)	155 (11.7)		105 (10.1)	125 (12.0)	
Moderate	19 (1.8)	26 (2.0)		19 (1.8)	21 (2.0)	
Severe	4 (0.4)	5 (0.4)		4 (0.4)	4 (0.4)	

## Discussion

We found that the time of surgery (morning or afternoon) did not affect both the overall incidence and severity of PONV. The association between PONV and time of day remained statistically insignificant prior to and after propensity matching for a number of covariates.

The pacemaker neurons in the suprachiasmatic nucleus of the hypothalamus regulate the 24-hour circadian rhythm in the human body. External factors like irregular sleep, artificial light and drugs can disturb this circadian control with profound consequences on cognition, organ physiology and even gene expression [3,12]. Although conditions like ST segment elevated myocardial infarctions have consistently been found to be highly correlated with time of day (more in early mornings than later in the day) [4,13], postoperative outcomes like mortality, morbidity and length of hospital stay have inconsistently correlated with the time of day [1, 14-18]. Kork et al., in a large retrospective study of 247,475 patients, found that all-cause in-hospital postoperative mortality was lower in operations done in the morning (8 am to 11 am) compared to those beginning in the afternoon (1 pm to 5 pm) [14]. Their results were similar to studies by Kelz et al. [19] and Whitlock et al. [20] which reported higher postoperative mortality in surgery starting after 4 pm. The authors postulated that both logistical factors (like healthcare provider experience and availability) and human physiology (like provider fatigue and circadian hormonal effect on patients' ability to recover after surgery [21-23]) could contribute to the circadian variation in outcomes. In contrast, recent retrospective studies failed to show any relationship between time of day and postoperative mortality [17, 18].

Most of the studies assessing the effect of time of day on postoperative outcomes have looked for associations with outcomes like mortality or length of hospital stay. To the best of our knowledge, there is only one published study (Wright et al.) which has assessed the effect of time of day on adverse anaesthesia outcomes like postoperative pain and PONV [2]. In their study, Wright et al. retrospectively analysed over 90,000 anaesthesia records at a tertiary hospital to determine the different frequencies of postoperative adverse events. They found that adverse events were more likely to occur after surgeries starting in the afternoon (12-3 pm) compared to those starting in the morning (9 am to noon) (Odds ratio, 0.88 (95% CI: 0.77 to 0.99)). Similar to the general analyses for adverse events, specific analysis for pain and PONV events also found an increased probability after surgeries starting late afternoon compared to those starting in the morning. The authors however did not provide any granular data like PONV risk score for the study population, frequency distribution of type of surgery, antiemetic usage, etc. to allow for a more objective assessment of their observations.

In our analysis, we not only compared characteristics like gender, race, laparoscopic surgery, intraoperative opioid use, and type of general anaesthesia between the morning and afternoon surgery groups but also matched for various confounders like session duration, surgical specialty, age at the time of surgery, and intraoperative antiemetics which were found to be statistically different between groups on univariate analysis. Although in both pre- and post-PSM analysis, we found a slightly higher absolute incidence of PONV in the afternoon surgery group compared to the morning surgery group, this percentage difference did not reach a statistical difference, in contrast to the study by Wright et al. [2].

This study has some limitations. Firstly, although our study analysed real-world data from a quality-controlled database and tried to propensity score match for many potential confounders which could affect the primary outcome, there could remain certain unbalanced unknown confounding factors which could affect the outcome. Therefore, a randomised control trial is recommended to further confirm results. Secondly, we were not able to analyse postoperative ward administration of rescue antiemetics, as it is yet to be integrated with the perioperative data in our analytical system. However, it is to be noted that all patients included in this analysis were assessed postoperatively by a member of the anaesthesia team and asked specific questions about the occurrence of PONV in the 24-hour postoperative period, so if a patient was to suffer from PONV serious enough to require rescue medication, it would have been captured in the postoperative review by our staff. Additionally, we chose 12 noon as the arbitrary cutoff for morning and afternoon surgery group definitions because we found no standard definition in similar previous studies. Lastly, we chose to exclude surgical sessions which extended across morning and afternoon from analysis because we wanted to avoid any carry-over effects that may arise from morning or afternoon circadian changes in body physiology that may affect the outcome.

## Conclusions

In conclusion, our analysis found that the time of surgery (morning or afternoon) did not affect both the overall incidence or severity of PONV. The association between PONV and time of day remained statistically insignificant prior to and after propensity matching for a number of covariates. Although our study tried to propensity score match for many potential confounders which could affect the primary outcome, there could remain certain unbalanced unknown confounding factors which could affect the outcome. Therefore, a randomised control trial is recommended to further confirm results.
